# Hoverfly (Diptera: Syrphidae) assemblage of an oak–hornbeam in the Merlino Wood Natural Reserve and implications for its conservation

**DOI:** 10.3897/BDJ.8.e54243

**Published:** 2020-07-29

**Authors:** Umberto Maritano

**Affiliations:** 1 Indipendent Researcher, Condove, Italy Indipendent Researcher Condove Italy

**Keywords:** Diptera, Syrphidae, Merlino Wood, hoverfly, Piedmont, Syrph the Net, *Spilomyia manicata*

## Abstract

Merlino Wood is a typical oak–hornbeam forest in the Po Plain hydrographic region. It is one of the few remaining lowland forests in Northern Italy and is a Regional Natural Reserve and a Site of Community Interest (code IT1160010). This is the first survey on hoverflies in the study area and they act as bioindicators to assess habitat conservation. Sampling was performed with three Malaise traps and an entomological net. A total of 61 species of Syrphidae were recorded between March and October 2019. Three of the species observed are considered to be under threat in Europe, three species have been recorded for the first time in Piedmont and *Heringia latitarsis* (Egger, 1865) has been recorded for the first time in Northern Italy. The Syrph the Net analysis has been used to assess habitat conservation. It shows good preservation of xylosaprophagous species, while the conservation conditions of other larval trophic categories are poor.

## Introduction

The Merlino Wood Natural Reserve (MWNR) is an oak–hornbeam forest located in the Po Plain hydrographic region (Northern Italy). It is a Regional Natural Reserve and a Site of Community Interest (SCI; code IT1160010). The oak–hornbeam woods are considered of high ecological value and, for this reason, are listed as a habitat of European interest according to Directive 92/43 EEC (Sindaco et al. 2003). Genovesi et al. (2014) consider the conservation status of this habitat in Italy to be bad and decreasing; there are very few woods of this kind remaining on the Po Plain, due to the considerable exploitation of this area. The remaining lowland woods are mostly located in Piedmont (Camerano et al. 2009) and only 38% of all oak-hornbeam woods scattered throughout this region fall within a protected area (Camerano et al. 2009). The most important in terms of extension or historical conservation are the following: “La Mandria” Natural Park in Venaria Reale, Stupinigi Natural Park, “Bosco delle Sorti della Partecipanza” Natural Park in Trino and the Merlino Wood Natural Reserve in Caramagna Piemonte.

The MWNR has been repeatedly investigated for its Coleoptera fauna. Buprestidae assemblage, for instance, reveals a typical fauna of the Po Plain woods with European chorology clearly predominant as opposed to that of Mediterranean origin (Curletti 1992). Carabidae were sporadically collected: Giachino (1992) found four species associated with primary forests. Dutto (2007) found many xylosaprophagous species, such as *Osmoderma eremita* Scòpoli, 1763. Additional data were provided by Evangelista and Cristiano (2013), who highlighted the need for further research using standardised methodologies. There are no data available for Diptera: Syrphidae. There is virtually no knowledge of hoverfly distribution in the forest habitat of Piedmont. We have only a small amount of sporadic data from 19^th^ century collections in the Natural Science Museum of Turin (Sommaggio 2007) and the Civic History Museum of Bra (Umberto Maritano, unpublished data) and the tags often fail to report the precise location of their sampling. Based on the scarce data available, the Piedmont plain must have been rich in Syrphidae species in the 19^th^ century. Except for sporadic unpublished data, the only regional monitoring in this taxonomic group is in an alluvial alder forest in Mareschi, a hamlet within the municipality of Sant’Antonino di Susa (Maritano and Sommaggio 2020). However, there are plenty of data to highlight the impoverishment of the lowland fauna in the Eastern Po Plain (Bertollo et al. 2012). Consequently, it is desirable to carry out a detailed study of some areas of the north-west plain.

In order to assess ecosystem conservation, it is very important to organise sampling activities to standardise monitoring (Speight 2012). Hoverfly larvae show evident ecological preferences, with even slight variations in habitat conservation being able to modify Syrphidae populations. For this reason, they can be used as a bioindicator (Sommaggio 1999, Burgio et al. 2015). The Syrph the Net (StN) database (Speight 2008) can be used to compare the species observed against the species that we expect to find (those species strictly related with the habitat assessed and present in that region) and so estimate the functional value of an ecosystem (Speight and Castella 2001). This research gathers data on the Syrphidae fauna of the Merlino Wood in Caramagna Piemonte (CN) and aims to assess the conservation conditions of this habitat.

## Material and methods

**Figure 1.**
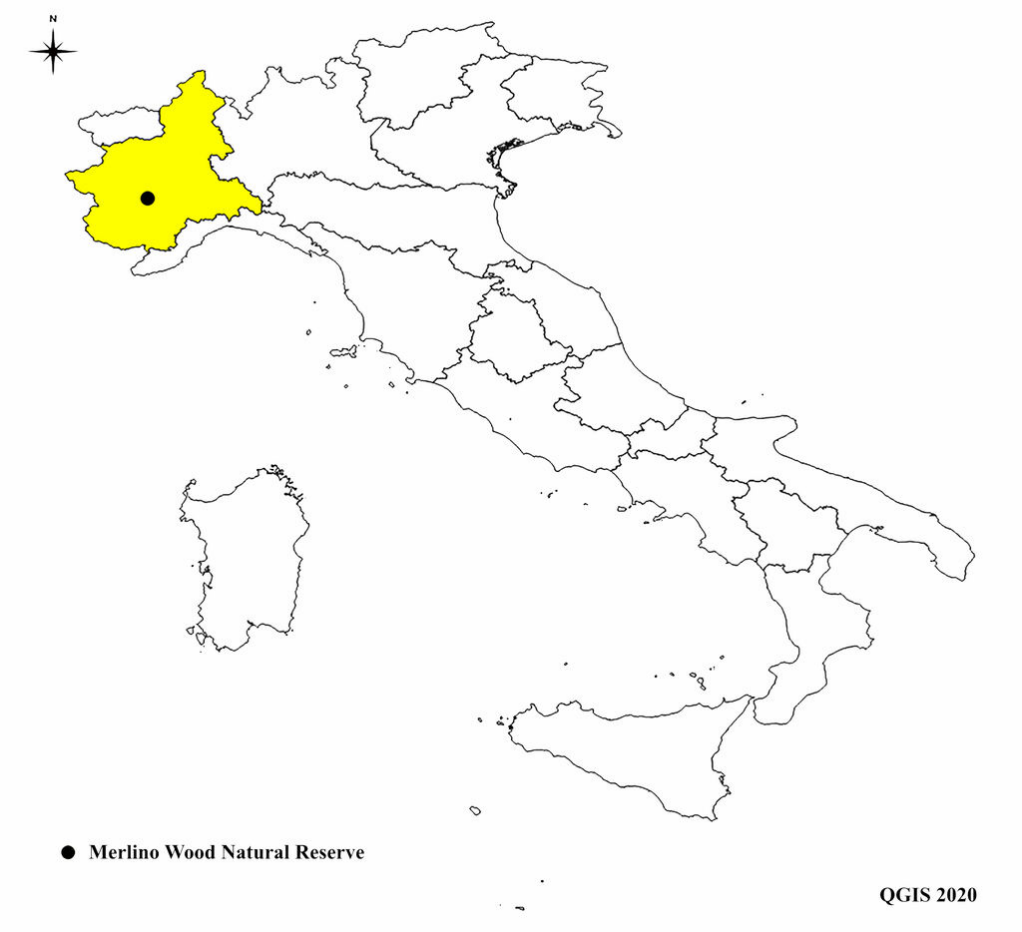
Position of Merlino Wood Natural Reserve in Italy, Piedmont Region in yellow.

**Figure 2.**
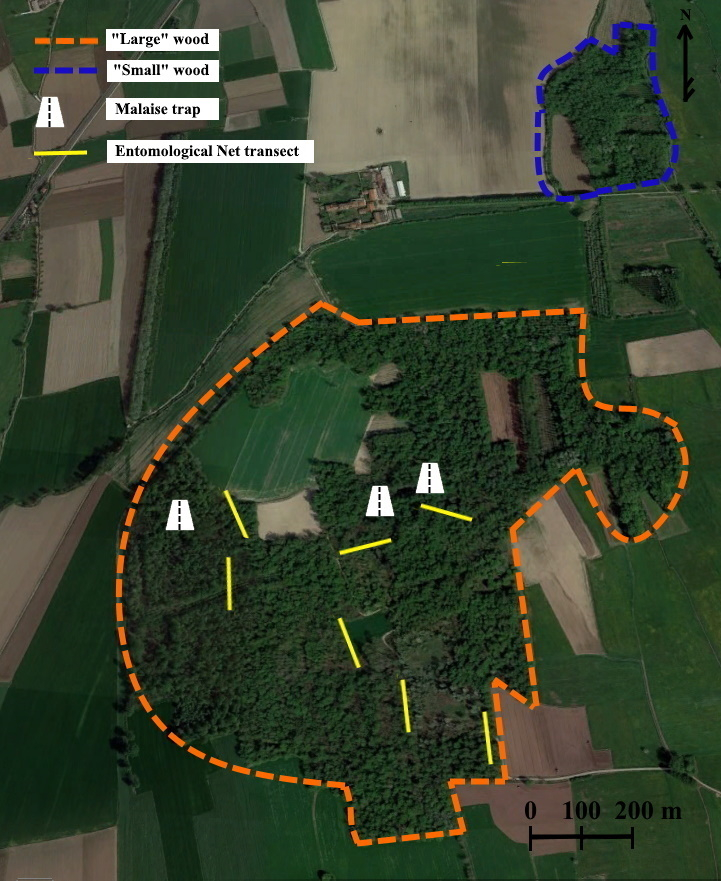
Merlino Wood and the sampling area.

Merlino Wood (Fig. 1) is spread over 49 ha (SCI Management Plan) and is located between Carmagnola and Caramagna Piemonte. Forest cover consists of 23% *Quercus robur* L., 1753, 40% *Carpinus betulus* L., 1753, 30% *Fraxinus excelsior* L. and very small areas of *Salix alba* L., 1753, *Populus tremula* L., *Ulmus minor* Mill., 1768, *Acer campestre* L., *Prunus avium* L., 1755, *Malus Sylvestris* Mill., 1768 and *Tilia cordata* Mill. (Abbà 1982, Dotta 1992). The site is divided into two subunits: one more extensive (large wood) and a smaller portion (Pica Wood or small wood). The sampling activities were concentrated in the central and southern portion of the large wood, over an area of approximately 25 ha (Fig. 2).

In order to include all phenological periods of adult Syrphidae species, field research began on 11 March and ended on 30 October 2019, using direct (entomological net) and passive (Malaise traps) collection methods. These two methods are thought to be complementary to collect adult hoverflies (Burgio and Sommaggio 2002). Entomological net sampling was performed once a week on clear, sunny days and each sampling lasted ca. 6 hours (08:30–12:30 and 14:30–16:30). Each transect was an average of 100 m long and took 20 minutes (repeatable in the case of seasonal blooms). Three Malaise traps (180 cm high × 180 cm long × 120 cm thick) were set inside *Quercus*–*carpinus* wood with 70% ethyl alcohol; the samples were collected approximately once a week. The locations of the traps were 44°47'37"N, 7°42'46"E, 44°47'39"N, 7°42'50"E and 44°47'36"N ,7°42'30"E. The specimens collected were partly pinned (reference collection) and partly preserved in 80° ethyl alcohol. All samples are kept in the author’s entomological collection with future deposition in a publicly accessible collection. The keys proposed in Van Veen (2010), Bertollo and Sommaggio (2012) and Speight and Sarthou 2015 have been used to identify the species, while the taxonomy follows Burgio et al. (2015).

The use of Syrph the Net (StN) as a tool to assess the natural conservation of ecosystems has been widely tested in Europe (e.g. Speight and Castella 2001, Burgio and Sommaggio 2007, Burgio et al. 2015, Maritano and Sommaggio 2020). The StN database is an important source, concerning the features of most European Syrphidae species (Speight 2012, Speight 2016). StN has also been used to elaborate the Biodiversity Maintenance Function (BDMF), understood as the relationship between species observed in the field and expected species in a given habitat based on the StN database (e.g. as detailed in Speight 2012). BDMF is a numeric parameter expressed as a percentage. If the value is low (< 50%), it means that the conservation conditions of the area are poor, otherwise they are good (50-74%) or very good/ excellent (> 75%). A regional checklist is required to elaborate the list of expected species. Since a checklist of Syrphidae from Piedmont is not yet available, the list has been created, based on the list used in Maritano and Sommaggio (2020) integrated with Birtele (2011) for the presence of *Doros destillatorius* Mik, 1885.

## Data resources

The study yielded 61 species sampled in the Merlino Wood Natural Reserve. Table 1 lists all the species collected, as well as other useful information. The following species were first recorded in this region: *Cheilosia gigantea*, *Ferdinandea ruficornis* and *Heringia latitarsis*. The latter is the first record for Northern Italy (Burgio et al. 2015). *Ferdinandea ruficornis* has been known from very few records on the Po Plain (Sommaggio 2017). Three species (*Ferdinandea ruficornis, Mallota fuciformis and Spilomyia manicata*) are threatened in Europe, as stated in the StN database (Speight 2016). Seven other species are decreasing in Europe (*Brachyopa bicolor*, *Caliprobola speciosa*, *Ceriana conopsoides*, *Criorhina floccosa*, *Milesia crabroniformis*, *Volucella inflata and Xanthogramma stackelbergi*), with all of them exhibiting a notable decrease in numbers of population and/or range within the geographical area concerned during the 20^th^ century (Speight and Castella 2015).

## Results

By applying Syrph the Net (including migratory species and using the Piedmont checklist as reference), the BDMF value in over-mature mesophilic *Quercus/Carpinus* habitat is 37.5% (Table 2). This denotes an ecosystem with poor conservation conditions (Speight 2012). There are nine non-migrant and unexpected Syrphidae species in the main macrohabitat. These species (observed, but not expected) are: *Caliprobola speciosa*, *Cheilosia gigantea*, *Eristalinus aeneus*, *Eristalinus sepulchralis*, *Merodon avidus*, *Paragus haemorrhous*, *Spilomyia manicata*, *Syritta pipiens* and *Xanthogramma stackelbergi*. Of these, five species are very common and rural and are probably distributed in the surrounding agricultural ecosystem. *C. speciosa* is a rare saproxylic species more associated with thermophilic oak forest (Speight 2016). *Cheilosia gigantea* is usually found in unimproved montane grassland (Speight 2016) and its presence here is surprising. The presence of *S. manicata* is very interesting, because it is generally associated with *Fagus* forest (Speight 2020) and, in Italy, is indicated in a very few scattered records.

Microhabitats associated with old trees seem to be in very good conservation conditions (Table 3). Phytophagus species and detritivores (terrestrial and aquatic saprophagus species) seem to be very deficient. Amongst larval microsite evaluation (Table 4), phytophagous species are poorly represented in stem bases and bulbs/tubers, due to few observed *Eumerus*, *Merodon* and *Cheilosia* species. Foliage is well represented because many predator species occupy this space. Hollow trees are a fundamental element in a forest ecosystem (Ulyshen 2018) and the data for Merlino Wood are very good (Table 4), considering that there were two species observed but not expected species in this category.

**Figure 3.**
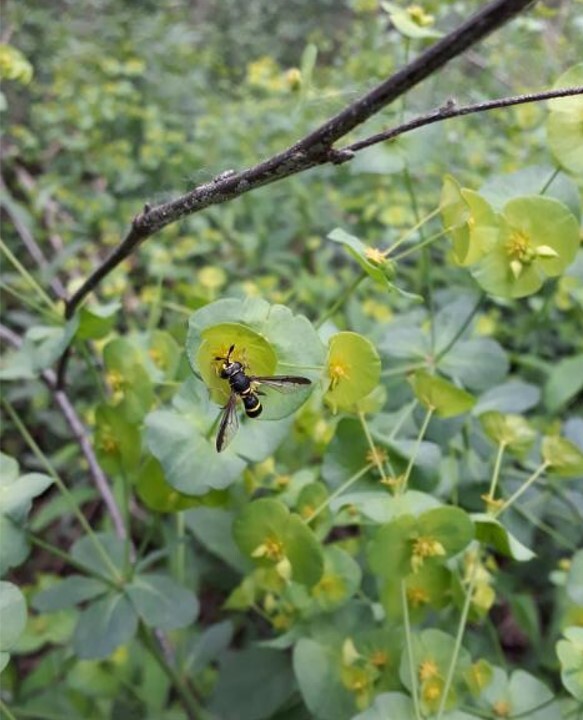
*Ceriana conopsoides* on *Euphorbia* L., in Merlino Wood Natural Reserve.

**Figure 4.**
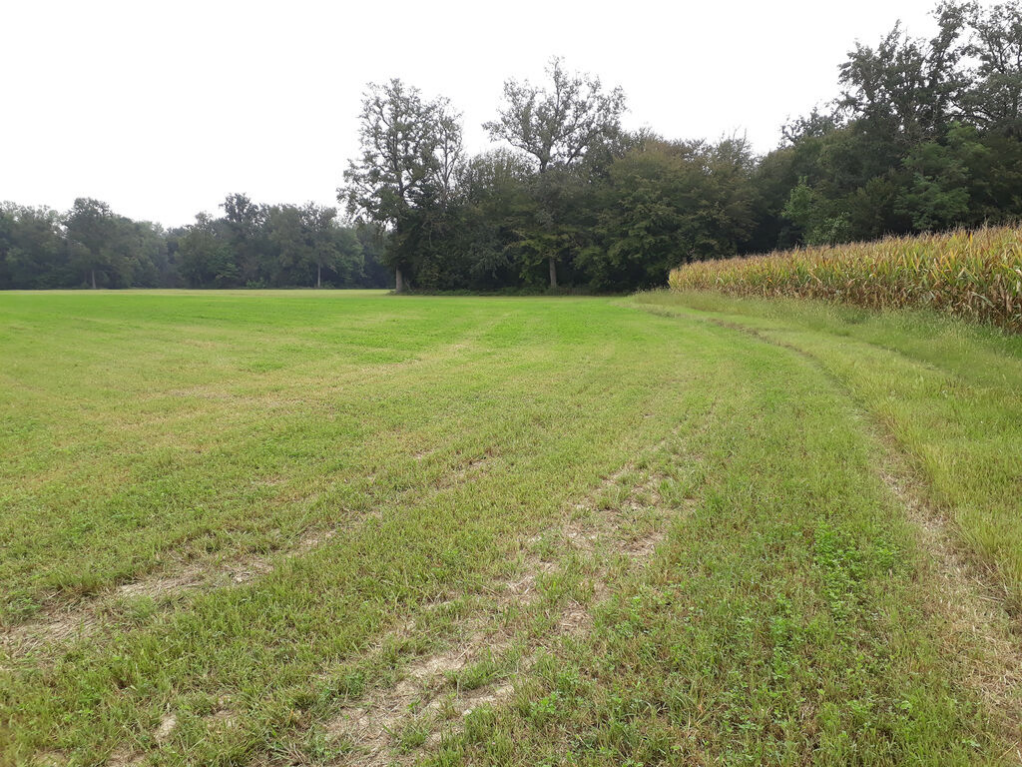
Typical ecotone at border to oaks of the Merlino Wood Natural Reserve.

Hoverflies are the main pollinators after Apoidea (Burgio et al. 2015) and are fundamental to functional diversity. In Merlino Wood, several (mostly rare) saproxylic species perform a primary action as pollinators in shrubs and herbaceous layers. *Prunus* sp. supports *Mallota fuciformis* and *Criorhina floccosa* adult flies; *Crataegus* sp. flowers were visited sometimes by *Caliprobola speciosa*. *Ceriana conopsoides* has been observed on flowers of *Euphorbia* sp. (Fig. 3). *Convolvolus* sp. blooms in clearings located near the forest were inspected by *Spilomyia manicata* within 50 m of the ecotone edge; on larger blooms of *Convolvolus* sp. located 1 km from the wood and sampled using the same methodology applied in the study area during the peak period of activity of the adult species, *S. manicata* was absent. *S. manicata* can probably travel for long distances only in the dispersion phase and not during regular daily movement while looking for flowers. Lastly, *Geranium nodosum* is often visited by very common hoverfly like *Episyrphus balteatus* and *Helophilus pendulus*, but is also sometimes visited by *Ferdinandea* sp. The presence of intensive crops in the ecotone (Fig. 4) strongly affects the availability of suitable blooms.

## Conclusions

Conservation actions on saproxylic insects play a key role in the management of forest ecosystems (Speight 1989), especially through operations of retention and restoration ecology (Ulyshen 2018, Samways et al. 2020). Oak woods in excellent conservation conditions show a considerable diversity of saproxylic species with reference to the hoverfly genera *Callicera*, *Ferdinandea*, *Mallota*, *Myolepta* and *Spilomyia* (Ricarte et al. 2009). Merlino Wood shows good diversity of saproxylic hoverfly, as revealed by the BDMF of 54%. However, a bottleneck effect can be assumed in relation to the hypothetical coenosis present when the forest fragment was connected to form an extensive plain network. This hypothesis is supported by historical data that show extensive distribution of lowland forest in Piedmont (Camerano et al. 2009). Merlino Wood currently seems completely isolated, although the possibility of biological exchanges with other lowland woods, such as Racconigi (3.5 km), Stupinigi (20 km) and Staffarda (24 km), should be considered. The total of 61 species found in the study area compared to the few other faunistic studies available (Birtele et al. 2002) for the same type of habitat can be considered similarly. There is, however, a shortage of extensive studies on Syrphidae assemblage in oak-hornbeam forest in Italy and particularly in Piedmont. Consequently, it is impossible to define the distribution of the species or their site evaluation. Merlino Wood can be considered a priority conservation area in Piedmont due to the high number of threatened or decreasing species and also because it offers us the only population record of *Spilomyia manicata* actually known in association with Oak-hornbeam woods in Europe (Speight 2020), making Merlino Wood unique.

From the conservation strategy viewpoint, it would be advisable to avoid planting intensive crops close to the forest. There should be a buffer zone of at least 50 m around the full whole perimeter of the wood, in addition to internal clearings, assuming harvesting at times that do not compromise the blooming of the grasses. This could also favour phytophagous species. Forest management should allow the possibility of indefinite growth of the oldest oaks, in order to support the greatest possible number of microhabitats. The average age of the oldest *Quercus robur* is just 110–120 years, which is too young to reach adequate senescence. By contrast, in *Quercus* woods in Slavonia (Croatia), the minimum shearing turn is 150–160 years and, in the French forest of Tronçais, it is 250 years (Dotta 1992). Moreover, actions should be taken to ensure controlled renewal of the oak and to limit alien species.

Merlino Wood is subject to enormous human pressure, but still represents an isolated source of saproxylic organisms. Strict habitat conservation measures are required to preserve its flora and fauna.

## Figures and Tables

**Table 1.** Checklist of Syrphidae sampled in the Merlino Wood Natural Reserve. Very low density indicates that only a single specimen was seen in the field during the entire season.

Faunistic list	Malaise trap (N of specimens)	Net (N of specimens)	First observation month	Very low density
*Baccha elongata* (Fabricius, 1775)	1		June	
*Brachyopa bicolor* (Fallen, 1817)	1		March	x
*Brachypalpoides lentus* (Meigen, 1822)	1	1	May	
*Brachypalpus laphriformis* (Fallen, 1816)		1	March	x
*Caliprobola speciosa* (Rossi, 1790)		1	April	x
*Ceriana conopsoides* (Linnaeus, 1758)		1	May	
*Chalcosyrphus nemorum* (Fabricius, 1805)	2		July	
*Cheilosia gigantea* (Zetterstedt, 1838)		1	May	x
*Cheilosia proxima* (Zetterstedt, 1843)		1	June	x
*Chrysotoxum bicinctum* (Linnaeus, 1758)		1	June	
*Chrysotoxum cautum* (Harris, 1776)	1	2	March	
*Chrysotoxum festivum* (Linnaeus, 1758)	1	1	May	
*Criorhina floccosa* (Meigen, 1822)		1	April	
*Criorhina ranunculi* (Panzer, 1804)		1	April	
*Dasysyrphus venustus* (Meigen, 1822)	2		April	
*Epistrophe melanostoma* (Zetterstedt, 1843)	1		April	
*Epistrophella euchroma* (Kowarz, 1885)	1		May	
*Episyrphus balteatus* (De Geer, 1776)	4	1	March	
*Eristalinus aeneus* (Scopoli, 1763)		3	March	
Eristalinus sepulchralis (Linnaeus, 1758)		1	June	
*Eristalinus taeniops* (Wiedemann, 1818)		1	August	
*Eristalis arbustorum* (Linnaeus, 1758)		3	June	
*Eristalis interrupta* (Poda, 1761)		1	March	
*Eristalis tenax* (Linnaeus, 1758)		3	March	
*Eumerus ornatus* Meigen, 1822		7	June	
*Eupeodes corollae* (Fabricius, 1794)	1	2	May	
*Eupeodes latifasciatus* (Macquart, 1829)	1	1	March	
*Eupeodes luniger* (Meigen, 1822)		2	May	
*Ferdinandea cuprea* (Scopoli, 1763)		4	May	
*Ferdinandea ruficornis* (Fabricius, 1775)		1	May	
*Helophilus pendulus* (Linnaeus, 1758)	17	1	March	
*Helophilus trivittatus* (Fabricius, 1805)		2	May	
*Heringia heringi* (Zetterstedt, 1843)		2	April	
*Heringia latitarsis* (Egger, 1865)		2	May	
*Mallota fuciformis* (Fabricius, 1794)		1	March	
*Melanostoma mellinum* (Linnaeus, 1758)		6	April	
*Melanostoma scalare* (Fabricius, 1794)	1	3	March	
*Merodon avidus* (Rossi, 1790)		3	May	
*Milesia crabroniformis* (Fabricius, 1775)		1	July	
*Myathropa florea* (Linnaeus, 1758)		2	April	
*Myolepta vara* (Panzer, 1798)		1	April	x
*Paragus haemorrhous* Meigen, 1822		4	April	
*Paragus pecchiolii* Rondani, 1857		5	April	
*Pipizella viduata* (Linnaeus, 1758)		12	March	
*Psilota anthracina* Meigen, 1822		1	May	x
*Scaeva dignota* (Rondani, 1857)		1	May	
*Scaeva pyrastri* (Linnaeus, 1758)		1	April	
*Sphaerophoria rueppelli* (Wiedemann, 1830)		2	July	
*Sphaerophoria scripta* (Linnaeus, 1758)		1	May	
*Spilomyia manicata* (Rondani, 1865)		1	June	
*Syritta pipiens* (Linnaeus, 1758)		2	March	
*Syrphus ribesii* (Linnaeus, 1758)	4	3	June	
*Syrphus torvus* Osten-Sacken, 1875		1	March	
*Syrphus vitripennis* Meigen, 1822	19	3	March	
*Volucella inanis* (Linnaeus, 1758)		1	July	
*Volucella inflata* (Fabricius, 1794)		1	May	
*Volucella pellucens* (Linnaeus, 1758)		1	June	
*Volucella zonaria* (Poda, 1761)		1	June	
*Xanthandrus comtus* (Harris, 1780)	20		March	
*Xanthogramma stackelbergi* Violovitsh, 1975	1	2	May	
*Xylota segnis* (Linnaeus, 1758)		1	May	x

**Table 2.** Derivation of BDMF (Biodiversity Maintenance Function) percentage value for macrohabitat, based on Syrph the Net (StN).

Macrohabitat	CORINE Code	StN Code	Expected species (N)	Observed species (N)	BDMF (%)	Unpredicted species observed not migrant (N)
Mesophilic Quercus/Carpinus over-mature	41.2	11221	112	42	37.5	9

**Table 3.** BDMF percentage value for trophic larval categories, based on Syrph the Net (StN). Exp.: Number of Expected species, Obs.: Number of Observed species

Macrohabitat	Xilosaprophagous			Phytophagous			Predators			Detritivores		
	Exp.	Obs.	%	Exp.	Obs.	%	Exp.	Obs.	%	Exp.	Obs.	%
Mesophilic Quercus/Carpinus over-mature	28	15	53.6	23	4	17.4	49	20	40.8	12	3	25

**Table 4.** BDMF percentage values for voltinism and larval microsites, based on Syrph the Net (StN).

		Expected species (N)	BDMF (%)
Number of generations/year	< 1	5	60.0
	1	71	28.2
	2	46	41.3
	> 2	10	70.0
Larval microsite	Foliage	25	48.0
	Stem bases	11	18.2
	Grass-root zone	14	50.0
	Bulbs/tubers	10	30.0
	Mature trees	33	48.5
	Timber stumps	9	55.6
	Timber fallen	4	75.0
	Timber standing	5	60.0
	Trunk cavities	17	58.8
	Sap runs/lesions	18	44.4
	Nests of social insects	5	60.0
	Submerged sediment/debris	8	37.5
